# Effects of photobiomodulation on human dental pulp stem cells treated with a bone xenograft: An in vitro study

**DOI:** 10.1007/s10103-026-04845-0

**Published:** 2026-03-13

**Authors:** Priscilla Pelaez-Cruz, Pia Lopez-Jornet, Eduardo Pons-Fuster

**Affiliations:** University of Murcia, Murcia, Spain

**Keywords:** Photobiomodulation, Dental pulp stem cells, Bone regeneration, Biocompatibility

## Abstract

Background: Biocompatibility between bone cells and biomred for the process of osteogenesis. The aim of this study was to assess the effect of Photobiomodulation (PBM) in vitro on dental pulp stem cells (DPSCs) treated with Bio-Oss® (BO) bone substitute xenograft. Methods: DPSC cultures were obtained from third molars extracted from healthy volunteers and characterized by flow cytometry. The effects of PBM (5, 7, or 10 J/cm^2^) on DPSCs treated with BO (5 mg/ml) were assessed using cytotoxicity assays, viability staining, cell migration, alkaline phosphatase activity, and Alizarin red S staining. Results: PBM restored the lost migration ability of DPSCs treated with BO; however, this combined therapy was associated with significantly lower levels of DPSC viability, alkaline phosphatase expression, and mineralization compared to controls. Conclusion: PBM therapy did not improve the biocompatibility of DPSCs treated with BO bone substitute. The effect of interaction between these treatments seemed to be cytotoxic to DPSCs. Our results suggest that the interaction between BO and PBM therapy could impair bone regeneration.

**Graphical abstract:**

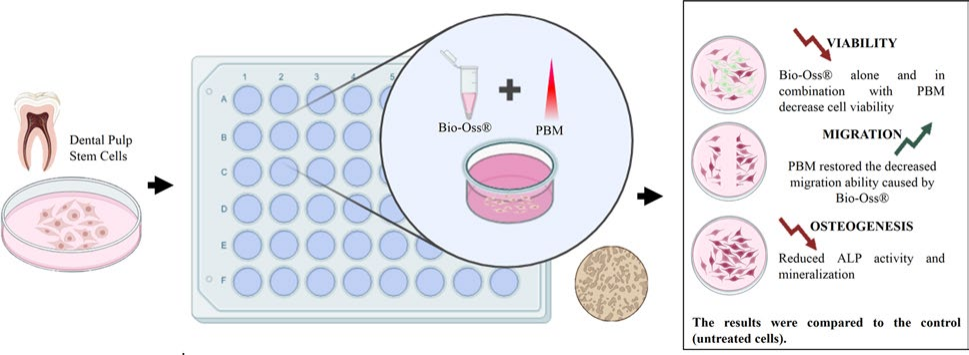

## Introduction

In recent decades, research has found that low-level laser therapy, also known as photobiomodulation (PBM), is able to promote tissue regeneration. Based on the use of low-power laser or light-emitting diode (LED) light with wavelengths of 500 to 1100 nm and powers of 1 to 500 mW, this therapy works by stimulating cell metabolism in the areas exposed, reducing inflammation, ameliorating pain, and promoting healing [1, 2].

The benefits of PBM have been widely exploited in dental treatments, this technique being considered effective both for soft and hard tissue. In particular, it has been used to treat conditions such as sensitive teeth, post-extraction dental pain, mucositis, and temporomandibular joint pain, as well as promote orthodontic tooth movement and stimulate bone regeneration [3]. In the field of odontology, bone defect repair still poses a challenge, and therefore there is great interest in the search for new strategies that would allow bone tissue regeneration [4].

Bone regeneration allows us to regain bone tissue volume and morphology in bone defects and it can be promoted through the use of biomaterials developed from various sources; and among them, xenografts are considered to be a good option due to their clinical safety and efficacy [5–8]. Bio-Oss® (Geistlich Pharma, Wolhusen, Switzerland) is a xenograft obtained from deproteinized cancellous bovine bone, which has similar physical and chemical properties to that of human bone. This biomaterial has osteoconductive and osteoinductive properties [9] and has shown to provide good clinical results in relation to bone regeneration in periodontal and dentoalveolar treatments [10–12]. On the other hand, previous in vitro studies have indicated that this bone substitute has an impact on the viability, proliferation, and migration of certain cell groups [13–16].

In vitro studies allow us to assess the biocompatibility and biological effects of biomaterials on the cell tissues before their use in clinical trials [17]. Mesenchymal stem cells (MSCs) derived from the oral cavity represent a valuable source for regenerative medicine due to their accessibility and differentiation potential [18]. This group includes several distinct cell populations, such as periodontal ligament stem cells (PDLSCs) and dental pulp stem cells (DPSCs), which play active roles in tissue repair and immunomodulation [19, 20]. In particular, DPSCs constitute a specialized population of oral-derived MSCs, exhibiting notable osteogenic potential and the ability to interact with biomaterial scaffolds, thereby supporting their application in bone tissue engineering and regenerative dentistry [21–24]. Their relative ease of isolation and minimal invasiveness, together with the ability to obtain sufficient cell numbers from limited tissue samples, make DPSCs an attractive and practical cell source for bone regeneration and biomaterial-based tissue engineering approaches [25, 26].

Several in vitro studies have demonstrated that PBM is able to stimulate biological reactions such as the proliferation and differentiation of various cell groups in culture, as well as reduce the activity of proinflammatory cytokines in cell cultures [27–30]. In this context, the aim of this study was to assess the in vitro effect of PBM on DPSCs treated with Bio-Oss® xenograft bone substitute.

## Materials and methods

### Cell isolation and culture

We obtained DPSCs from third molars extracted from three healthy volunteers between 18 and 40 years old. The study was approved by the ethics committee (ID: 3729/2021), and all participants gave written informed consent. Several vials of cells were frozen as a reserve prior to testing. Cell culture passages 3–6 were used in the experiments.

Dental pulp was cut into small pieces with a scalpel and subsequently disaggregated in a collagenase (3 mg/mL) and dispase (4 mg/mL) solution for 1 h at 37 °C. The digested extracts were strained (through a 70-μm filter) and cultured on a 100-mm cell culture plate. Cell cultures were expanded in complete cell culture medium (alpha-MEM medium supplemented with 10% FBS, 400 mM of penicillin/streptomycin, and 2.5 μg/ml of amphotericin B) and incubated at 37 °C in a 95% oxygen and 5% carbon dioxide mixture.

For alkaline phosphatase activity assays and Alizarin red S staining, we used an osteogenic medium (complete medium supplemented with 50 μl/ml ascorbic acid, 7.5 mM β-glycerol phosphate, and 1 μM dexamethasone) to induce cell differentiation.

### DPSC characterization

The mesenchymal phenotype of the cells isolated was confirmed by analyzing the expression of mesenchymal markers using flow cytometry (Human Mesenchymal Stem Cell Multi-Color Flow Kit- FMC002- R&D SYSTEMS).

The cell samples were washed with 2 ml of staining buffer and centrifuged at 300 g for 5 min. Subsequently, 1.5 × 10^6^ cells in 1 ml of staining buffer were transferred into a 5 ml cytometry tube. Then, 10 μl of four fluorochrome-conjugated antibodies (CD105, CD46, CD90 and CD45) and their corresponding isotype controls, were added to the cells. The mixture was then incubated for 45 min at room temperature in the dark. After incubation, the cells were washed with 2 ml of staining buffer, centrifuged, and resuspended in 400 μl of staining buffer for flow cytometry analysis.

**Fig. 1:**
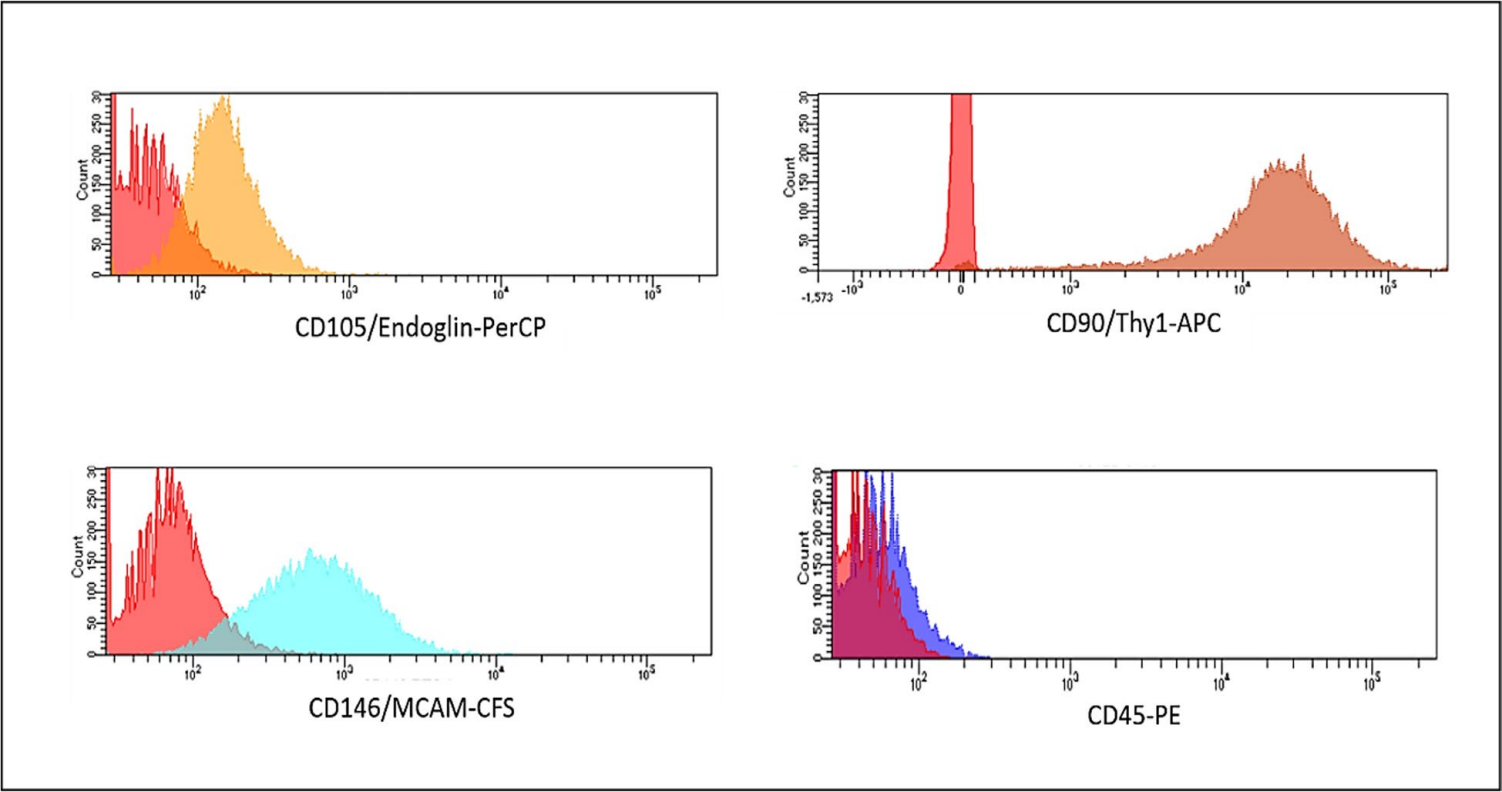
Characterization of dental pulp stem cells

Over 95% of the cells were positive for the mesenchymal markers CD105, CD46, and CD90, and negative for CD45 (Fig. 1).

### Preparation of bone substitute

Bio-Oss® (BO) (Geistlich Pharma AG., Wolhusen, Switzerland) 0.25–1 mm particles (Batch: 82,001,136) were weighed with a precision balance in a laminar flow chamber and used as a dilution (5 mg/ml, incubated for 24 h in complete medium).

### Photobiomodulation

For PBM, we used an Epic™ 10 diode laser (Biolase, CA, USA), emitting at 940 nm in continuous mode and with an output power of 0.2 W, applying an energy density of 5 J/cm^2^ for 25 s, 7 J/cm^2^ for 35 s, or 10 J/cm^2^ for 50 s (hereon referred to PBM5, PBM7, and PBM10, respectively). The DPSCs seeded with a bone substitute were irradiated once using the “surgical handpiece” (diameter 0.5 cm) at a standard distance of 1 mm above the top of the well plates under dark conditions.

### Evaluation of DPSC viability/proliferation – the MTT assay

Cells were cultured in 96-well plates seeded at 5000 cells/cm^2^ with 200 μl of complete culture medium, and 24 h after incubation, they were treated with PBM5, PBM7, PBM10 and BO alone or in combination with PBM5, PBM7, or PBM10. The results were quantified at two different incubation times (48 and 72 h). After the incubation period, the medium was replaced with a 1 mg/ml solution of MTT ((3-(4, 5-dimethylthiazol-2-yl)−2, 5-diphenyltetrazolium bromide), and cells were incubated for 4 h. Then, MTT was removed from the well and the formazan, a product of cell metabolism, was dissolved in 100 μl of dimethyl sulfoxide. Lastly, absorbance was measured with a spectrophotometer at a wavelength of 570 nm and a reference wavelength of 690 nm.

### Evaluation of DPSC viability – Viability staining assay

DPSCs were cultured as for the MTT assay (in 96-well plates, seeded at 5000 cells/cm^2^, and with 200 μl of complete culture medium). After 24 h of incubation, cells were treated with PBM5, PBM7, PBM10 and BO alone or in combination with PBM5, PBM7, or PBM10. The results were assessed at two different incubation times (48 and 72 h), using a Viability/Cytotoxicity Assay Kit for Animal Live & Dead Cells (Biotium, Fremont, CA, USA), according to the instructions of the manufacturer. After the incubation period, cells were rinsed with PBS to remove serum esterase activity, and the solutions of calcein AM and ethidium homodimer III (Eth-DII) contained in the kit were added. After being held for 30 to 45 min at room temperature, the stains were replaced by PBS, and cells were observed using an inverted fluorescence microscope.

### Evaluation of cell migration

DPSCs were cultured in 48-well plates (5000 cells/cm^2^ in 400 μl of complete culture medium) and incubated until monolayer confluence. Next, some areas of the cell monolayer were “wounded” (scraped away) with the sterile tip of a micropipette, the medium was replaced and the cultures were treated with PBM5 and BO alone or in combination with PBM5. Cell migration was quantified by taking photos of the damaged areas at 0 and 48 h of incubation using an inverted microscope with a digital camera (Nikon Eclipse TE2000-U, Nikon, Japan). The distance between the edges of the damaged area in the cell monolayer was measured by pixel counting using the ImageJ digital image processing software (National Institutes of Health [NIH], Maryland, USA), and the following equation was applied: migration distance = cell-free initial distance—cell-free distance after 24 h.

### Evaluation of alkaline phosphatase enzyme activity

Cells were cultured as for the cell migration assay (in 48-well plates seeded at 5000 cells/cm^2^ with 400 μl of complete culture medium) and incubated for 72 h. Subsequently, the medium was replaced by an osteogenic medium, and cells were treated with PBM5 and BO alone or in combination with PBM5.

The activity of alkaline phosphatase was assessed using a commercial kit (Alkaline Phosphatase Activity Fluorimetric Assay Kit; Sigma-Aldrich, St. Louis, MO, USA) according to the manufacturer’s instructions. In this kit, ALP cleaves the phosphate group of the non-fluorescent substrate of the disodium salt of 4-methylumbelliferyl phosphate (MUP), producing a strong fluorescent signal. The ALP activity is measured by the amount of fluorescence emitted in each treatment.

After 7 days of culture, cells were lysed in a 1% Triton X-100 solution (Sigma-Aldrich, St. Louis, MO, USA) for 30 min at 37 °C. The cells from each treatment were transferred to Eppendorf tubes and centrifuged at 13,000 G for 3 min. Subsequently, 5 μL of the supernatant from each treatment and 105 μL of the ALP Buffer solution containing the kit were added to a 96-well fluorescence plate. Four replicates were placed for each treatment (S) and a duplicate of each one as a control (SBC). Then, 20 μL of stop solution was added to the SBC wells to stop alkaline phosphatase activity. Additionally, 20μL of the MUP substrate at 0.5 mM was added to all S and SBC wells.

A standard curve (ST) was also prepared according to the manufacturer's instructions, using concentrations of 0, 0.1, 0.2, 0.3, 0.4, and 0.5 nmol per well.

All preparations (S, SBC, and ST) were then incubated for 30 min at 25 °C, protected from light and fluorescence (relative fluorescence units, RFU) was measured in a FLUOstar Omega plate reader (BMG Lab. Technologies, USA), with an excitation wavelength of 360 nm and an emission wavelength of 440 nm.

### Evaluation of in vitro biomineralization potential - Alizarin red S staining

DPSCs were cultured as for the cell migration and ATP activity assays (in 48-well plates seeded at 5000 cells/cm^2^ with a 400 μl of complete culture medium) and incubated for 3 days. Next, the medium was replaced by an osteogenic medium, and cells were treated with PBM5 and BO alone or in combination with PBM5. For this assay, as well as the positive control (untreated cells cultured in osteogenic medium), there was a negative control (untreated cells cultured in complete control medium). The medium was replaced every 3 days throughout the experimental study (21 days).

After the culture period, cells were rinsed with PBS, fixed with 4% formaldehyde, and treated with Alizarin red S (VWR International, Barcelona, Spain) at 2% to stain the nodules of calcium formed in each of the samples yellowish orange. Subsequently, photographs were taken of all the wells using an inverted microscope equipped with a digital camera (Nikon Eclipse TE2000-U).

### Statistical analysis

Data were examined by performing analysis of variance and Tukey's multiple comparison tests using GraphPad Prism v7.0 (GraphPad Software Inc, San Diego, USA). The threshold for statistical significance was set at *p* = 0.05 (95% confidence interval).

## Results

### Effects on DPSC viability/proliferation – MTT assay

MTT assays were carried out to determine the proliferation and viability of DPSCs as well as the cytotoxicity to these cells when treated with BO alone or in combination with PBM at one of three energy densities (PBM5, PBM7, or PBM10), through the quantification of metabolic activity.

**Fig. 2:**
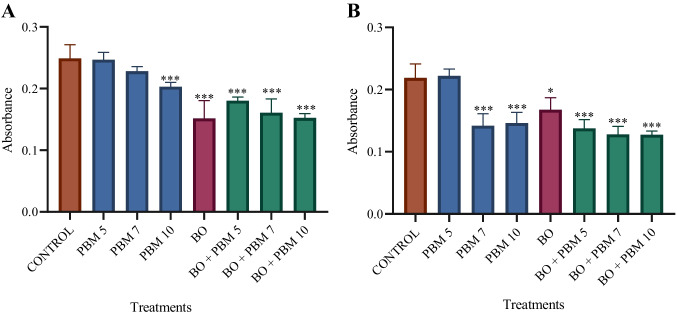
Effect of photobiomodulation (PBM), at a dose of 5 J/cm2 (PBM5), 7 J/cm2 (PBM7), or 10 J/cm2 (PBM10), on the viability of dental pulp stem cells (DPSCs) treated with Bio-Oss (BO) after 48 h (A) and 72 h (B) of culture. The data showed that for both durations of cell culture treatment with BO alone and in combination with any of the doses of PBM was associated with a significant decrease in viability with respect to that in the control group. The results are presented as mean ± standard deviation *P < 0.05, **P < 0.01, ***P < 0.001

The results after 48 h (Fig. 2A) and 72 h (Fig. 2B) of culture indicated that cells treated with BO both alone and in combination with PBM5, PBM7, or PBM10 had significantly lower cell viability than control group cells (untreated cells cultured with complete medium). Considering PBM therapy alone, we also observed that differences in cell viability at 48 h with respect to controls were not significant with PBM5 or PBM7, while viability was significantly lower with PBM10. Further, after 72 h of culture, cell viability after treatment with either PBM7 or PBM10 alone was significantly lower than in controls, while differences did not reach significance in the case of PBM5.

### Effects on DPSC viability – Viability staining assay

**Figure3:**
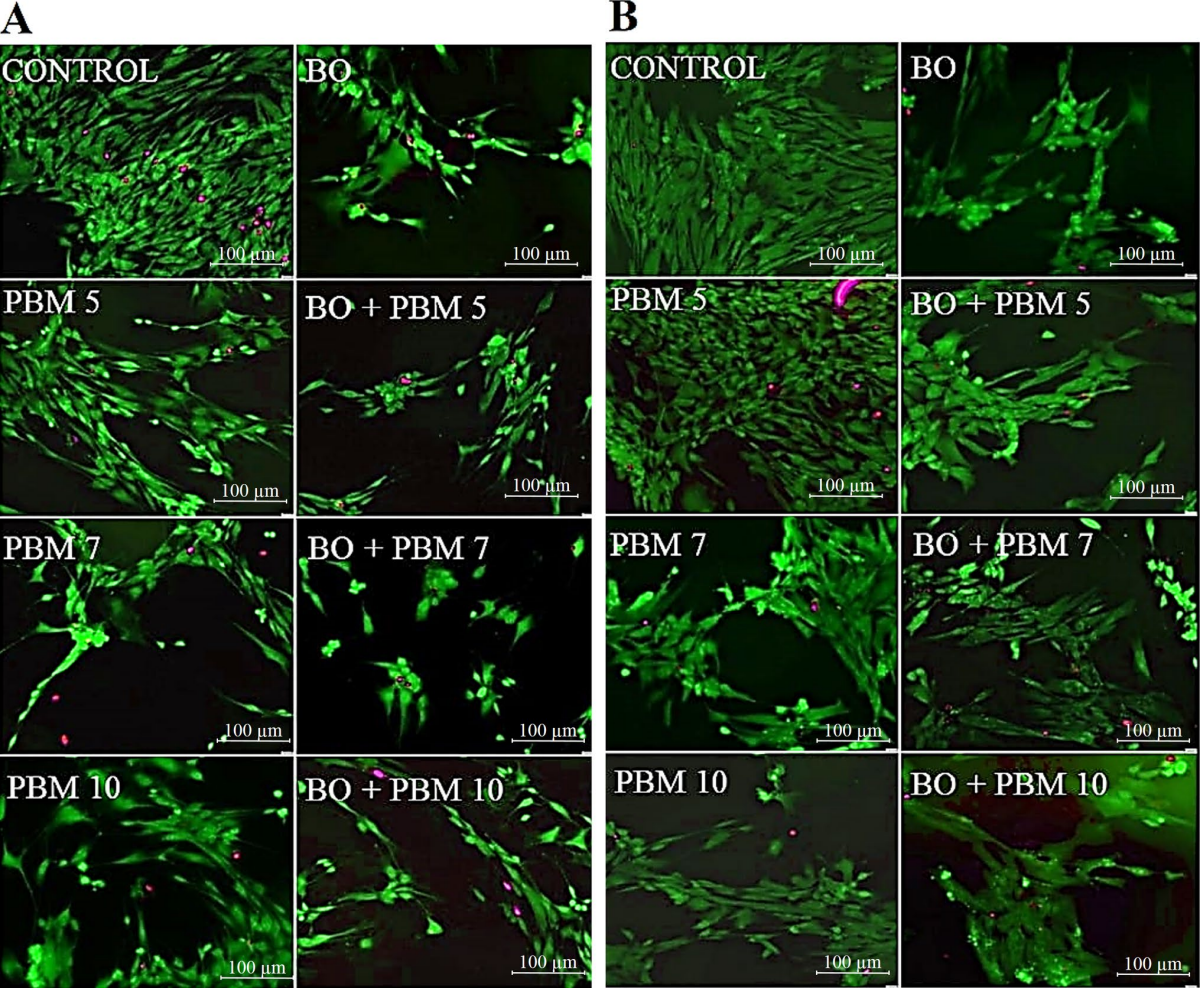
Fluorescence microscopy images of dental pulp stem cells (DPSCs) treated with Bio-Oss (BO) alone or in combination with photobiomodulation (PBM), at a dose of 5 J/cm2 (PBM5), 7 J/cm2 (PBM7) or 10 J/cm2 (PBM10), after 48 h (A) and 72 h (B) of culture. Live cells are stained green, while red staining indicates cells with damaged membranes

Viability staining enabled us to qualitatively identify the viability of DPSCs treated with BO both alone and in combination with PBM5, PBM7, or PBM10, after the corresponding incubation time, staining viable cells green and dead cells red. In the images obtained with this assay after 48 h (Fig. 3A), we observed that cultures with BO contained fewer viable cells than control cultures (untreated cells cultured with complete medium). We also observed that although cells treated with PBM5, PBM7, or PBM10 had lower cell confluence than controls, the reduction in confluence was more marked when PBM was applied in combination with BO.

Further, after 72 h of culture (Fig. 3B), as after 48 h, we found that cultures with BO contained fewer viable cells than control cultures. In this case, the results were similar for PBM7 and PBM10 alone and in combination with BO. In contrast, with PBM5, a similar number of cells were observed to that in controls when PBM therapy was applied alone, while cell confluence was notably lower when PBM5 was used in combination with BO.

### Effects on cell migration

**Fig. 4:**
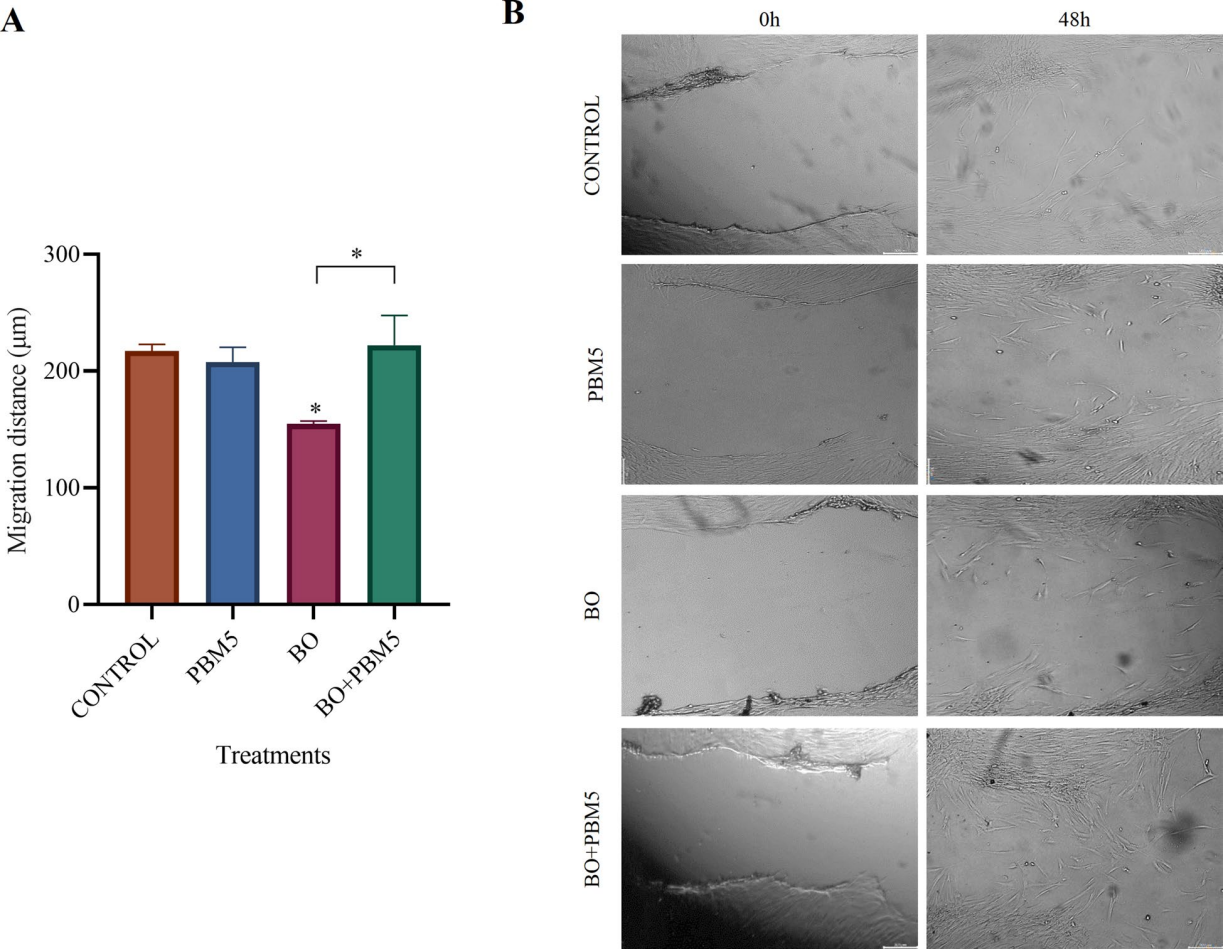
Effect of photobiomodulation at a dose of 5 J/cm2 (PBM5) on the migration ability of dental pulp stem cells (DPSCs) treated with Bio-Oss (BO) after 48 h of culture. Differences between PBM5 and control treatments were not significant. Use of BO alone was associated with significantly lower migration ability while this negative effect was reversed when BO was used in combination with PBM5. The results are presented as mean ± standard deviation *P < 0.05, **P < 0.01, ***P < 0.001

The migration ability of DPSCs treated with BO alone or in combination with PBM5 was assessed using the wound healing assay after 48 h of culture. In this assay (Fig. 4), the use of BO alone was associated with DPSCs showing significantly lower migration ability compared to controls (untreated cells cultured with complete medium), while when used in combination with PBM5, this negative effect was reversed. Nonetheless, migration observed using PBM5 alone was not significantly different from that in controls.

### Effects on alkaline phosphatase enzyme activity

**Figure5:**
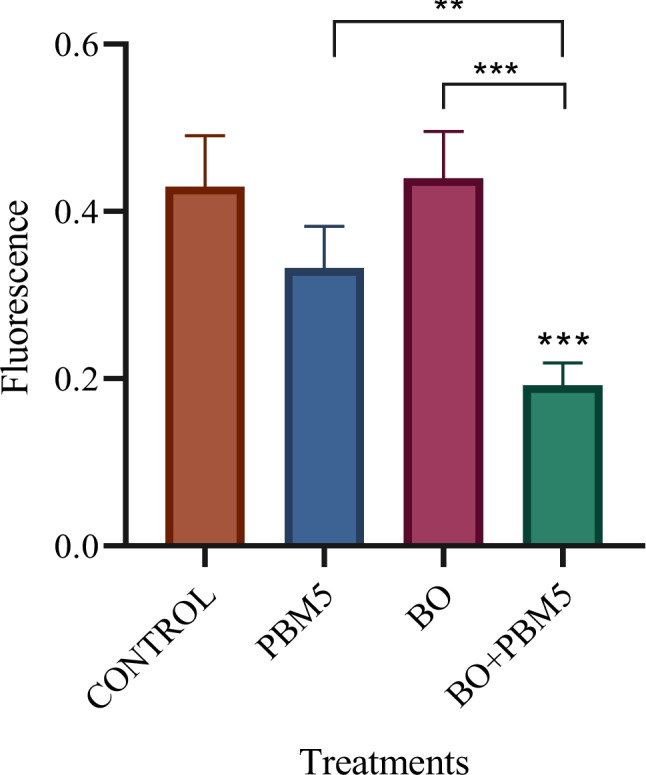
Effects of photobiomodulation at a dose of 5 J/cm2 (PBM5) on alkaline phosphatase activity in DPSCs treated with Bio-Oss (BO) after 7 days of culture. The level of activity observed did not differ significantly from that in controls when BO or PBM5 were used alone, while their combination was associated with significantly lower alkaline phosphatase expression than in control cultures, as well as in cultures with BO or PBM5 alone. Results are expressed as mean ± standard deviation *p < 0.05, **p < 0.01, ***p < 0.001

After 7 days of culture, differences compared to controls were not statistically significant with BO or PBM5 alone (Fig. 5). On the other hand, in the cells treated with BO in combination with PBM5, ALP expression was significantly lower than in positive control group cells (PC), as well as in those treated with BO or PBM5 alone. On the other hand, no ALP expression was observed in cells of the negative control (NC, untreated cells cultured with complete medium).

### Effects on in vitro biomineralization potential - Alizarin red S staining

The mineralization ability of DPSCs treated with BO alone or in combination with PBM5 was qualitatively assessed after 21 days of culture. For this, we used Alizarin Red S staining that enabled us to visualize calcium nodules formed in each of the samples in yellowish orange.

**Fig. 6:**
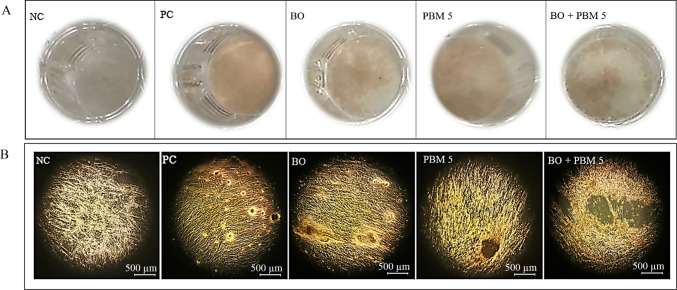
Effects of photobiomodulation at a dose of 5 J/cm2 (PBM5) on the mineralization of dental pulp stem cells (DPSCs) treated with Bio-Oss (BO) after 21 days of culture. A. Alizarin red S staining in each treatment, B. Images of the calcium deposits stained with Alizarin red S in each treatment. Treatment with BO was associated with lower mineralization ability, as was BO in combination with PBM5, while with PBM5 alone, the extent of staining was similar to that in the positive control (PC). Negative control (NC) cells were not stained

This staining indicated that the mineralization ability of DPSCs treated with BO was lower than that of controls (Fig. 6). The results were similar to those found in the cells treated with BO in combination with PBM5. On the other hand, we observed similar staining with PBM5 alone and the PC, while no staining was observed in cells of the NC (untreated cells cultured with complete medium).

## Discussion

In this study, we explored the effect of the PBM therapy on DPSC cultures treated with the BO xenograft using: cytotoxicity assays, viability staining, measurements of cell migration and ALP activity, and Alizarin red S staining (for assessing bone mineralization).

Biocompatibility, defined as the ability of a material to perform its function with an appropriate immune response from the host, is one of the key characteristics required for biomaterials with respect to the process of osteogenesis. In line with this, bone cells must be able to adhere to, migrate, proliferate, and differentiate when in contact with bone substitutes [13, 31, 32].

The results of the MTT assay after 48 and 72 h of culture indicated that both BO alone and in combination with PBM at doses of 5, 7, or 10 J/cm^2^ (i.e., PBM5, PBM7, or PBM10) had a possible cytotoxic effect on DPSCs. Moreover, the results of this cytotoxicity assay were consistent with those of viability staining, in which we observed fewer cells than in controls after treatment with BO alone and in combination with PBM5, PBM7, or PBM10. Further, in the MTT and viability staining assays, PBM5 was the only dose at which cell viability was not compromised after the culture durations assessed (48 and 72 h). Therefore, only this dose of PBM was used in the other assays (cell migration, ALP activity, and Alizarin red S).

Our results regarding BO are in line with those of Rombouts et al. (2016) and Jeanneau et al. (2020) in periodontal ligament cells, Ayobian-Markazi et al. (2012) and Bernhardt et al. (2011) in osteoblast phenotype cells, and Herten et al. (2009) in human osteoblast and bone marrow stem cells, in which BO decreased the viability of the aforementioned cell types in culture. Mladenović et al. (2013) reported that BO, even at low concentrations, is able to reduce levels of calcium and phosphorus ions in a cell culture medium. As it is known that the presence of these ions is key to the proper functioning of bone cells, this would explain our results with this biomaterial.

Further, the effects of PBM in vitro have been assessed in various studies, showing that it is able to promote cell proliferation, migration, and mineralization. On the other hand, research has also found that its effects on cell metabolism may vary depending on the radiation protocol and cell type assessed [27, 30, 36–38].

Studying DPSC cultures placed on magnesium-based bioceramic scaffolds with and without PBM treatment at 2 or 4 J/cm^2^, Theocharidou et al. (2017) did not find significant differences in viability after 1, 2, or 3 days of culture. These results differ from ours and this may be attributed to differences in the methodology, biomaterials, and radiation protocols used.

On the other hand, our results on cell migration indicated that the migration ability of cells lost after treatment with BO was restored following PBM5 therapy after 48 h of culture. Indeed, results in the cells treated with PBM5 did not differ significantly from those in controls.

To our knowledge, no previous studies have assessed DPSC cell migration using PBM therapy alone or in combination with biomaterials. Our results are similar to those of Paschalidou et al. (2020) who reported that cell migration after applying PBM using an energy density of 4 J/cm^2^ was not significantly different from that under control conditions in stem cells from exfoliated deciduous teeth after 24, 72, and 120 h of culture. Further, Shingyochi et al. (2017) found comparable results in human fibroblasts treated with PBM at 5 J/cm^2^ after 6, 12, and 24 h of culture.

Finally, the influence of PBM5 on osteogenic activity in DPSCs treated with BO was assessed through ALP activity assays and Alizarin red S staining. ALP is considered an initial marker of hard tissue formation and cell differentiation. Our results after 7 days of culture showed no significant differences compared to controls when BO and PBM were used separately, while when combined, the expression of ALP was significantly lower than in controls. As in our study, Amid et al. (2022) found that ALP activity did not differ significantly between DPSCs exposed to PBM at 5 J/cm^2^ and controls after 14 or 28 days.

Alizarin red S staining allowed us to qualitatively identify the formation of calcium nodules in DPSCs after 21 days of culture following the treatments under study. In the images obtained, BO in combination with PBM5 therapy was associated with less staining than in controls, that is, with a lower mineralization ability.

Research has previously indicated that the implantation of biomaterials, as for any other foreign material, can lead to inflammation in the host tissue. Such inflammation is considered a key factor for activating bone regeneration, given that it promotes the release of growth factors and cell differentiation. This process is mainly triggered by the release of reactive oxygen species (ROS) by macrophages and neutrophils around the implantation site. In general, cells have a mechanism to neutralize low levels of ROS. Nonetheless, certain factors such as the composition of biomaterials and products released during their reabsorption may increase ROS levels, leading to a redox imbalance and oxidative stress that compromise cell functions [41–43].

On the other hand, it has been noted that the mechanism of action of PBM therapy is based on the absorption of laser light by the cytochrome c oxidase mitochondrial chromophore, and in turn, the promotion of several processes, such as increases in blood flow, ATP production and release of ROS to stimulate the tissue repair [44–48]. It has been reported that the higher the dose of radiation, the greater the release of ROS [46, 48]. This would also explain the possible cytotoxic effect of PBM7 and PBM10 observed in our MTT and viability staining assays.

As described above, ROS play key roles in cell functions and biocompatibility between cells and bone substitutes. The results of our study indicate that the lowest dose of PBM (PBM5) alone did not have an impact on viability, ALP activity, or cell migration, compared to controls. Nonetheless, in combination with BO, it seemed to affect cell biocompatibility. Given previous research indicating that both biomaterials and PBM can trigger ROS release in host tissue, we believe that the adverse effects on cell biocompatility observed in our study may have been the result of an additive effect of the two treatments, this leading to ROS production above physiological levels tolerated by cells, and as a consequence, an increase in cell death.

The energy densities used in this study (5, 7, and 10 J/cm^2^) were selected a priori based on the *Arndt–Schulz* law, which describes the biphasic dose–response behavior commonly observed in PBM. According to this principle, low energy densities promote cellular stimulation, whereas higher doses may lead to reduced or inhibitory biological responses [49, 50]. This framework has been widely used to guide the selection of PBM parameters in experimental studies, particularly in vitro models. Additionally, dose response studies in mesenchymal stem cells, including dental derived stem cell populations, have reported biostimulatory effects across a range of low energy densities, with optimal values varying depending on cell type and experimental conditions [1, 2, 27, 30, 36, 51]. Therefore, the selected energy densities were chosen to explore a biologically relevant window and to assess potential dose dependent responses in xenograft-treated DPSCs.

Taking into account the limitations of this study, particularly its in vitro design and the limited availability of comparable studies, the results should be interpreted within the context of these experimental conditions. In static cell culture environments, metabolic byproducts and potential cytotoxic agents may accumulate, leading to biological effects that could be attenuated under physiological conditions [52]. In contrast, in vivo environments benefit from vascularization, which facilitates the continuous clearance of waste products and ROS, as well as a dynamic immune response and the buffering capacity of body fluids [53, 54]. Consequently, the cytotoxic effects observed in the present study may be less pronounced within a complex biological system. Nevertheless, in vitro models provide a controlled experimental setting to investigate specific cellular mechanisms and interactions between photobiomodulation and biomaterials [55]. Therefore, further studies employing in vivo and clinically relevant models are warranted to confirm the translational relevance of these findings and to optimize regenerative therapies for the treatment of bone defects.

## Conclusion

Our results show that PBM therapy did not improve the biocompatibility of DPSCs treated with the bone substitute Bio-Oss. The effect of the interaction between these treatments seemed to be cytotoxic to DPSCs.

## Data Availability

No datasets were generated or analysed during the current study.

## Declarations

The research followed the principles of the Declaration of Helsinki and institutional guidelines.

### Clinical trial number

Clinical trial number: not applicable.
